# Clinical Implications of the Transversus Abdominis Plane Block in Adults

**DOI:** 10.1155/2012/731645

**Published:** 2012-01-19

**Authors:** Mark J. Young, Andrew W. Gorlin, Vicki E. Modest, Sadeq A. Quraishi

**Affiliations:** ^1^Department of Anesthesiology, Critical Care and Pain Medicine, Massachusetts General Hospital, Boston, MA 02114, USA; ^2^Department of Anesthesiology, Perioperative and Pain Medicine, Brigham and Women's Hospital, Boston, MA 02115, USA; ^3^Harvard Medical School, Boston, MA 02115, USA

## Abstract

The transversus abdominis plane (TAP) block is a relatively new regional anesthesia technique that provides analgesia to the parietal peritoneum as well as the skin and muscles of the anterior abdominal wall. It has a high margin of safety and is technically simple to perform, especially under ultrasound guidance. A growing body of evidence supports the use of TAP blocks for a variety of abdominal procedures, yet, widespread adoption of this therapeutic adjunct has been slow. In part, this may be related to the limited sources for anesthesiologists to develop an appreciation for its sound anatomical basis and the versatility of its clinical application. As such, we provide a brief historical perspective on the TAP block, describe relevant anatomy, review current techniques, discuss pharmacologic considerations, and summarize the existing literature regarding its clinical utility with an emphasis on recently published studies that have not been included in other systematic reviews or meta-analyses.

## 1. Introduction

The transversus abdominis plane (TAP) block is a regional anesthesia technique that provides analgesia to the parietal peritoneum as well as the skin and muscles of the anterior abdominal wall [1]. First described just a decade ago, it has undergone several modifications, which have highlighted its potential utility for an increasing array of surgical procedures [2]. Despite a relatively low risk of complications and a high success rate using modern techniques, TAP blocks remain overwhelmingly underutilized [3]. Although the block is technically straightforward, there is inertia regarding its adoption into clinical practice. In part, this may be related to limited sources for anesthesiologists to develop a comprehensive understanding of the transversus abdominis plane. As such, we provide a brief historical perspective on the TAP block, describe relevant anatomy, review current techniques, discuss pharmacologic considerations, and summarize the existing literature regarding its clinical utility. 

## 2. History

**Figure 1:**
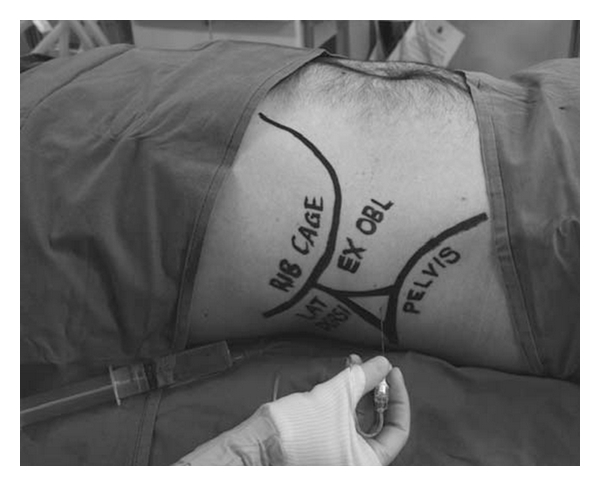
Surface anatomical landmarks can be utilized to identify the triangle of Petit [9]. Reproduced with permission.

Rafi first described the TAP block in 2001 [2]. He portrayed it as a refined abdominal field block, with a targeted single shot anesthetic delivery into the TAP, a site traversed by relevant nerve branches. This was a significant advance from earlier strategies that required multiple injections [4]. In this approach, utilizing surface anatomical landmarks, the TAP was reached by first identifying the lumbar triangle of Petit (Figure 1), an area enclosed medially by the external oblique, posteriorly by the latissimus dorsi, and inferiorly by the iliac crest [2]. A 24-gauge, blunt-tipped, 2-inch needle was then advanced perpendicular to the skin through a preceding skin incision until a single confirmatory “pop” was appreciated. This sensation was thought to indicate proper needle depth for anesthetic delivery. In 2004, McDonnell et al. presented preliminary work on TAP blocks in cadavers and in healthy volunteers at the scientific meeting of the American Society of Anesthesiologists [5]. Although referred to as the regional abdominal field infiltration (RAFI) technique, the authors brought forward preliminary evidence to support the anatomical basis for TAP blocks and demonstrated sensory loss spanning the xiphoid to the pubic symphysis following delivery of local anesthetic to the TAP via the triangle of Petit. By the time the study was completed and published in 2007, McDonnell and his colleagues had already adopted the term TAP block and had demonstrated its analgesic utility in patients undergoing open retropubic prostatectomy [6–8].

## 3. Anatomy

**Figure 2:**
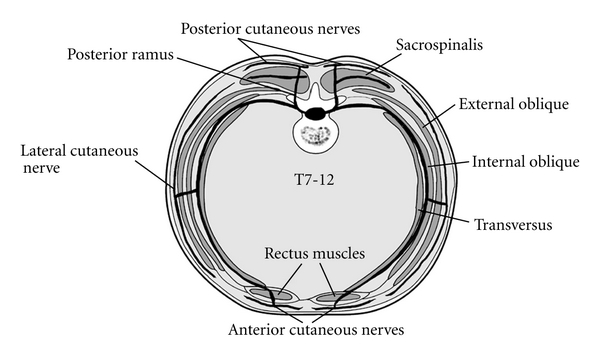
Transverse section of the abdominal wall demonstrating the relevant muscular structures and course of nerves (T7-T12) within the TAP [9]. Reproduced with permission.

**Figure 3:**
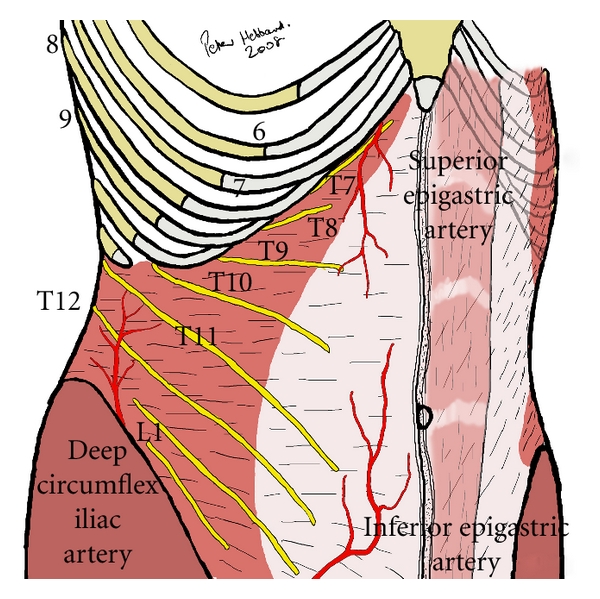
Typical distribution of nerves in the TAP. Generously shared from the personal files of Prof. P. Hebbard.

The musculature of the lateral abdomen has three layers (Figure 2). From superficial to deep, they are the external oblique, the internal oblique, and the transversus abdominis muscles. On its course from medial to lateral, the internal oblique muscle slopes upward and creates a small gap above the iliac crest. It is this sloping edge, above the iliac crest, that defines the medial aspect of the lumbar triangle of Petit (Figure 1). Based on cadaveric dissections, Jankovic et al. noted that the location of the medial edge of the triangle varies significantly between individuals, but is always located at a point posterior to the midaxillary line [10]. The posterior edge of the triangle is the latissimus dorsi muscle. It is not uncommon for the triangle to be quite small or poorly defined. Often, the external oblique may overlap the medial edge of the latissimus dorsi muscle. The inferior aspect of the triangle is the iliac crest, and the peritoneum rests directly deep to the innermost muscle. The TAP is the fascial layer between the internal oblique and the transversus abdominis muscles. It exists as a continuous plane located at any point on the abdomen where the two innermost muscle layers exist. Anterior rami of thoracolumbar nerves that innervate the anterior abdominal wall pass through this plane as small, but well-defined neurovascular bundles. Furthermore, Rozen et al. described an extensive fascial layer, nonadherent to the deep surface of the internal oblique that bind down the nerves on its deep surface, superficial to the transversus abdominis muscle [11]. They also observed that, while nerve segments from T6-L1 reliably innervate the abdominal wall, individual nerve segments branch and communicate extensively with other nerve segments as they travel in the TAP. Moreover, they noted that nerve segments entered the TAP from the costal margin in an inferolateral distribution such that segments from T6 entered adjacent to the linea alba whereas segments from T9 entered near the anterior axillary line (Figure 3). Along the anterior axillary line, between the costal margin and the ileum, near the triangle of Petit, nerves running in the TAP originate strictly from T9-L1.

## 4. Technique

### 4.1. Anatomical Landmark-Based Approaches

In Rafi's classic description of the TAP block, surface anatomic landmarks were used to determine the needle insertion site within the lumbar triangle of Petit, and a single “pop” sensation served as an endpoint for appropriate needle depth [2]. Patients were placed in the supine position, and a finger was walked from the anterior superior iliac spine along the top of the iliac crest until it dipped slightly inward. On further posterior movement, the finger tip was felt to slip over the lateral border of the latissimus dorsi, where it is attached to the external lip of the iliac crest. At this location, the skin was first pierced anterior to the finger tip with an 18-gauge cutting needle at the level of the external lip, and then followed by a 24-guage, blunt-tipped, 2-inch needle, which was inserted perpendicular to the skin until it touched the bone of the external lip. The needle was then slowly advanced over the intermediate zone of the iliac crest until the definite “pop” was felt. This single “pop” method differs from the “double pop” method described by O'Donnell et al.in which the needle was inserted cephalad to the iliac crest and advanced until two distinct “pops” were appreciated [8]. The authors explained that a “double pop” resulted from the blunt needle passing through the “fascial extensions of the abdominal wall muscles (external and internal obliques) within the floor of the triangle of Petit [12].” All anatomical landmark-based approaches to the TAP make use of blunt-tipped needles to improve tactile sensitivity and appreciation for distinct “pop” sensations.

### 4.2. Ultrasound-Guided Approaches

**Figure 4:**
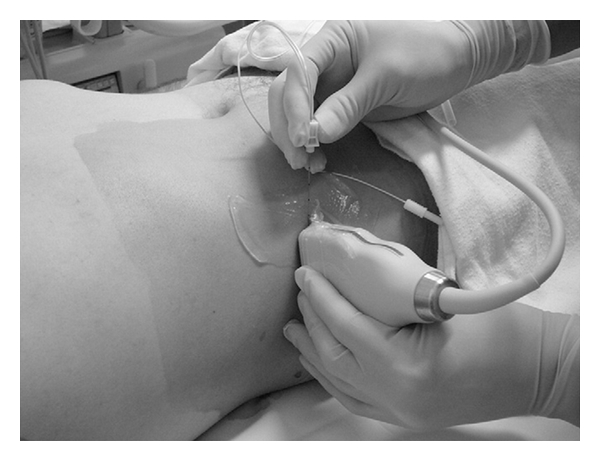
Ultrasound-guided TAP block showing needle alignment and ultrasound transducer placement on skin using an in-plane technique [20]. Reproduced with permission.

**Figure 5:**
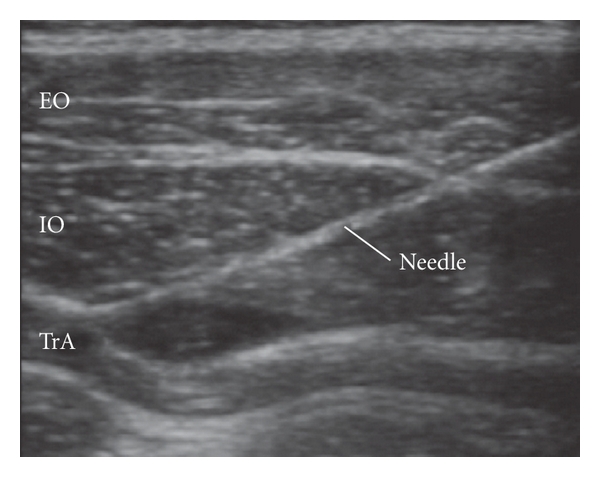
Ultrasound image during initial injection of a small amount of local anesthetic [9]. Reproduced with permission. EO: external oblique, IO: internal oblique, TrA: transversus abdominis.

**Figure 6:**
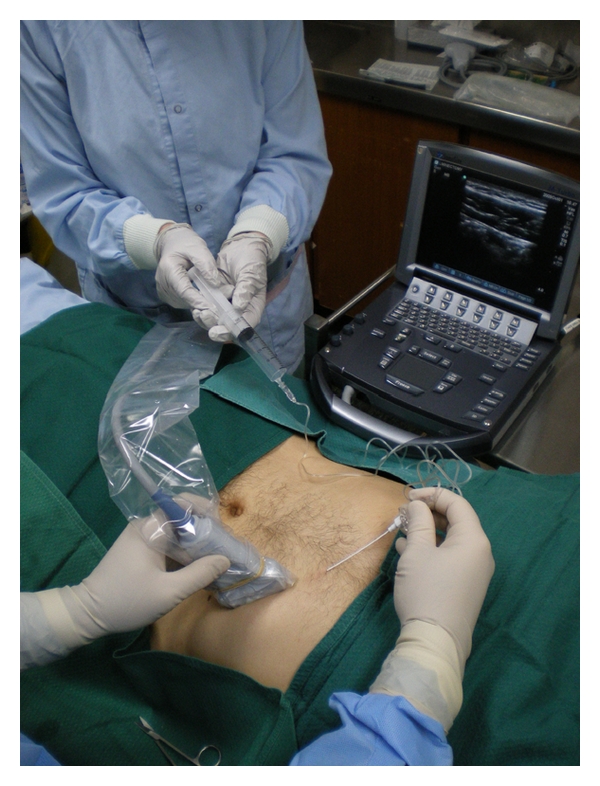
Placement of ultrasound probe for subcostal TAP blocks. Generously shared from the personal files of Prof. P. Hebbard.

An ultrasound-guided approach was first described in 2007 by Hebbard et al. [13]. The authors applied a transversely orientated ultrasound probe to the anterolateral abdominal wall where the three muscle layers are most distinct. After identification of the TAP between the internal oblique and transversus abdominis muscles, the probe was moved posterolaterally to lie across the midaxillary line just superior to the iliac crest (i.e., over the triangle of Petit). The block needle was then introduced anteriorly and advanced in an in-plane approach (Figure 4). Real-time ultrasonography facilitates easy needle visualization as it approaches and reaches the target fascial plane. A hypoechoic layer, created by injection of local anesthetic, is also easily visualized (Figure 5). Hebbard et al. also noted that the “pop” sensations in the classic approach could be imprecise due to anatomic variability, especially in patients with large BMI and, as such, concluded that real-time visualization of local anesthetic spread was likely to be a more definitive endpoint, as is often the case with other regional block techniques. This ultrasound-guided technique is commonly referred to as the posterior approach. In 2008, Hebbard described another ultrasound-guided TAP block technique designed for upper abdominal surgery referred to as the oblique subcostal approach [14]. In this variation, the needle entered the skin in an area near the xyphoid and was advanced inferolaterally such that local anaesthetic is delivered to the TAP along the costal margin (Figure 6). Importantly, the lateral abdominal muscle layers give way to an aponeurosis medially so that the TAP is defined by different muscle layers in this area. In some patients, the transversus abdominis muscle extended medially, and the roof of the TAP was formed by the rectus abdominis muscle. In other patients, the transversus abdominis muscle did not extend to the site of local injection, so the plane between the rectus abdominis and the rectus sheath was targeted. Børglum et al. recently described an ultrasound-guided, four-point, single-shot technique that combines the posterior and oblique subcostal techniques in an effort to provide wider bilateral analgesic coverage [15]. The subcostal TAP block was performed in a manner similar to that described by Hebbard when the transversus abdominis extended medially beneath the rectus abdominis [14]. This method was referred to as the medial intercostal TAP block. When the transversus abdominis terminated laterally at the linea semilunaris, the subcostal block was instead performed within the TAP at the lateral most extent of the transversus abdominis. This method was referred to as the lateral intercostal TAP block. In addition, the posterior TAP block was performed between the costal margin and the iliac crest at the anterior axillary line. It is important to note that the triangle of Petit is posterior to the midaxillary line, and, as a consequence, the posterior TAP as described in this study was performed in a location anterior to the original description [2].

### 4.3. Surgeon-Assisted Approaches

While the majority of published literature on TAP blocks is purely from the perspective of anesthesiologists, a growing number of reports have demonstrated that surgeons can help to facilitate these blocks. Chetwood et al. described a laparoscopic-assisted technique wherein a classic TAP block (based on anatomical landmarks) was performed while the injection area is observed with an intra-abdominal laparoscopic camera [16]. A peritoneal bulge at the area of injection was seen after local anesthetic was delivered within the TAP, and this visual served as the desired endpoint for this technique. Such direct visualization may help to avoid intraperitoneal injection, one of the major potential risks of the TAP block. More recently, a surgical TAP block utilizing a transperitoneal approach was also described. Performed intraoperatively, a blunt-tipped block needle was advanced from inside the abdominal wall through the parietal peritoneum, then the transversus abdominis muscle, and into the TAP as indicated by a single pop sensation [17, 18]. In addition, Araco et al. described a surgical TAP block in which blunt dissection through the external and internal oblique muscles leads to injection of local anesthetic into the TAP under direct visualization [19].

## 5. Local Anesthetic Dosing

In his original report, Rafi described the use of 20 mL of “a local anaesthetic agent” for each side requiring analgesia [3]. Subsequently, McDonnell et al. reported the use of 20 mL of 0.5% lidocaine for each side in healthy volunteers [5].  Table 1 provides a current summary of the various agents and related doses used in published clinical studies.

While local anesthetic agent, volume, concentration, and delivery method differ between studies, these regimens have not yet been compared against each other. Therefore, there is insufficient evidence to support any particular combination in lieu of another. When duration of analgesia is an issue, there is good evidence to support using TAP catheters. This technique was first described in 2009 in a small case series [42]. Two years later, the same group showed similar pain control between epidural and TAP catheter analgesia in a randomized study [26]. In both reports, an intermittent bolus protocol was used. It remains unclear whether the use of a continuous infusion offers any advantage over intermittent blousing for TAP catheters.

## 6. Clinical Use

TAP blocks have been described as an effective component of multimodal postoperative analgesia for a wide variety of abdominal procedures including large bowel resection, open/laparoscopic appendectomy, cesarean section, total abdominal hysterectomy, laparoscopic cholecystectomy, open prostatectomy, renal transplant surgery, abdominoplasty with/without flank liposuction, and iliac crest bone graft [8, 15–19, 21–48]. Most reports demonstrate the efficacy of TAP blocks by highlighting some combination of reduced postoperative opioid requirement, lower pain scores, and/or reduction in opioid-related side effects.

Petersen et al. reviewed 7 randomized, double-blinded, clinical trials of both landmark-based (*n* = 3) and ultrasound-guided (*n* = 4) TAP blocks for managing postoperative pain after abdominal surgery with incisions below the level of the umbilicus [49]. All 7 studies compared pain-related outcomes with TAP blocks as part of a multi-modal postoperative analgesic regimen. Morphine PCA ± acetaminophen ± nonsteroidal anti-inflammatory drugs was most commonly used to complement TAP blocks. In one study, intrathecal morphine was also part of the analgesic regimen. A meta-analysis of these 7 studies (180 cases and 184 controls) demonstrated an average reduction in 24-hour morphine consumption of 22 mg (95% confidence interval: −31 mg to −13 mg) in favor of TAP block patients compared with standard management. Furthermore, TAP blocks were associated with reduced early postoperative visual analog scores (VAS) both at rest and during mobilization in 4 of the 7 studies (1 study did not record VAS scores). Postoperative sedation, as well as postoperative nausea and vomiting (PONV), was marginally reduced in patients with TAP blocks. In a separate meta-analysis using 4 of the 7 studies reviewed by Petersen et al., Siddiqui et al. also demonstrated a morphine-sparing effect of TAP blocks in the first 24 hours after surgery [20]. Similarly, another meta-analysis by Charlton et al., which reviewed 236 participants from 5 studies (including landmark- and ultrasound-guided TAP blocks), demonstrated a significant reduction in 24-hour morphine requirements (average –22 mg, 95% confidence interval –38 mg to –6 mg) in TAP block patients compared to controls [1]. A significant difference in postoperative sedation, nausea, and vomiting was not appreciated between TAP-block and non-TAP block patients in this paper.

A number of new clinical studies utilizing TAP blocks have recently been published. Bharti et al. randomized 40 patients undergoing colorectal surgery to standard treatment (diclofenac and intravenous morphine) and bilateral intra-operative TAP block with either 0.25% bupivacaine (*n* = 20) or saline (*n* = 20) [17]. The bupivacaine group had a significant reduction in 24-hour morphine requirements (6.45 ± 3.26 mg versus 17.55 ± 5.78 mg; *P* < 0.0001) as well as a significant reduction in early postoperative pain scores both at rest and with coughing. Furthermore, early postoperative sedation scores were significantly lower in the bupivacaine group, and patient satisfaction was higher (6.8 ± 1.1 mg versus 3.5 ± 1.5 mg; *P* < 0.001). Although there was no difference between groups in the incidence of PONV, patients in the control group experienced significantly more severe PONV, requiring pharmacological intervention. Hivelin et al. studied the effect of TAP blocks for postoperative analgesia in patients with abdominal deep inferior epigastric perforator flaps for breast reconstruction [23]. The TAP block group (*n* = 15) required significantly less morphine (median and interquartile range: 28 mg (27 mg–38 mg) versus 42 mg (36 mg–46 mg); *P* = 0.0057) than controls (*n* = 15) in the first 24 hours after surgery. Early postoperative numerical pain scale scores were also significantly lower in the TAP block group compared to the non-TAP-block patients. However, no difference was observed between groups for postoperative sedation, PONV, and 48-hour satisfaction with pain management. Sforza et al. also studied the effect of TAP blocks on patients in the first 12 hours following abdominoplasty and reported significant postoperative morphine sparing, improved pain scores, and earlier ambulation in the TAP block group (*n* = 14) versus controls (*n* = 14) [21]. It is important to note that both groups received 10 mg of morphine and 1 gm of acetaminophen intraoperatively. Unfortunately, the information from this study is difficult to interpret and/or generalize, since data is reported without standard deviations or confidence intervals, and the follow-up time was abbreviated. However, not all reports demonstrate an analgesic benefit to TAP blocks when compared to standard therapy. Griffiths et al. randomized 65 patients undergoing surgery for presumed gynecologic malignancy to standard treatment (parecoxib, acetaminophen, and morphine) plus ultrasound-guided TAP block with either ropivacaine (*n* = 32) or saline (*n* = 33) [33]. No significant difference was found in the two groups for 24-hour morphine consumption (34 mg ± 27 mg versus 36 mg ± 27 mg; *P* = 0.76), VAS scores at rest (18 mm ± 19 mm versus 23 mm ± 22 mm; *P* = 0.33), VAS scores with coughing (39 mm ± 24 mm versus 48 mm ± 31 mm; *P* = 0.2), patient satisfaction (9 ± 2 versus 8 ± 3; *P* = 0.36), or incidence of nausea and pruritis. The authors speculated that the negative study may have been due to a combination of factors including a high incidence of obesity in the study population leading to potentially more technical failures, a wide age range, and the fact that 18 of the 65 patients had incisions that extended above the umbilicus (7 in the sham group versus 11 in the treatment group). The authors also hypothesized that the study population had a larger variation in “surgical insult;” that is, some cases involved more organ manipulation and dissection resulting in more visceral pain, for which TAP blocks would be less effective than those for parietal/incisional pain.

In another study, Baaj et al. randomized 40 women to receive either local anesthetic (*n* = 20) or saline (*n* = 20) TAP blocks in addition to a plain bupivacaine spinal block for elective cesarean section [30]. A significant reduction in 24-hour morphine requirement was observed in the local anesthetic TAP block group versus controls (26 mg ± 5 mg versus 63 mg ± 5 mg; *P* < 0.05). Although the authors report lower PONV, lower 24-hour VAS scores, and higher satisfaction in the local anesthetic TAP block group, no statistical measures were reported. These results are in line with previous reports by McDonnell et al. and Belavy et al. that demonstrated superior analgesia and significantly decreased 24-hour morphine consumption following C-section in patients who received TAP blocks in addition to plain local anesthetic spinal blocks when compared to patients with just local anesthetic spinal blocks [43, 48]. The use of intrathecal morphine, on the other hand, may minimize the analgesic advantage of TAP blocks when performed in addition to neuraxial blockade. McMorrow et al. randomized 80 patients to 4 equal groups (*n* = 20 in each arm) and reported that they found no overall analgesic advantage to TAP blocks and no incremental benefit of adding TAP blocks when patients receive intrathecal morphine [24]. They also reported similar overall patient satisfaction among groups despite more frequent pruritis in patients who received intrathecal morphine. These data are difficult to interpret since medians are reported without interquartile ranges (though box plots with whiskers are displayed) and comparisons are only performed between the highest and lowest scores in each category. Costello et al. also evaluated the incremental benefit of TAP blocks in addition to intrathecal morphine for C-section and found no difference in morphine requirements, VAS scores, or satisfaction in 96 patients (all received intrathecal morphine but were randomized to receive either local anesthetic or saline TAP blocks) [40]. Of note, the authors did not assess rates of nausea or pruritis. Similarly, Kanazi et al. compared TAP block without intrathecal morphine (*n* = 30) to sham TAP block with intrathecal morphine (*n* = 30) for c-sections and found a significantly longer time to first analgesic request as well as lower immediate postoperative VAS scores in the intrathecal morphine group [35]. However, the authors also reported significantly higher rates of both nausea and pruritis in the intrathecal morphine group as compared to the TAP block group. Patient satisfaction scores were similar between groups. Again, the data are difficult to interpret since only group medians and ranges are reported.

Few studies have compared TAP blocks to epidural analgesia. Recently, Kadam and Moran conducted a retrospective matched case-control study comparing continuous TAP block catheters (posterior and subcostal approaches; *n* = 15) to thoracic epidural analgesia (*n* = 15) [22]. Except for assessments in the postanesthesia care unit, there was no appreciable difference in pain scores between the two groups over a 3-day follow-up period. While patient satisfaction was similar between groups, the TAP block group required a significantly higher amount of breakthrough fentanyl over the study period. Therapeutic failure rate was higher in the epidural group (patchy block in 4 patients) versus the TAP catheter group (unilateral block in 2 patients). Hypotension was reported in 2 patients from the epidural group. As with a number of the previously discussed reports, these findings are difficult to interpret since the data tables do not provide sufficient information to perform a critical appraisal. In their prospective analysis, Niraj et al. compared continuous thoracic epidural analgesia to bilateral intermittent-bolus subcostal TAP catheters in open hepatobiliary and renal surgery patients [26]. The authors observed no significant difference in patient satisfaction as well as VAS scores at rest or with coughing at 8 hours to 72 hours after surgery. Rescue analgesia with tramadol, however, was significantly higher (*P* = 0.002) in the TAP catheter group (400 mg, interquartile range 300–500 mg) versus the epidural group (200 mg, interquartile range 100–350 mg) over 72 hours. Although a similar therapeutic failure rate was reported among the groups (22% versus 30% for epidural versus TAP), it should be noted that 8 of the TAP catheter patients had incisions or drains in locations not necessarily covered by subcostal TAP blocks. Data comparing hemodynamic consequences were not reported.

A number of case reports have also highlighted new potential clinical scenarios to integrate the use of TAP blocks. Singh et al. demonstrated that bilateral TAP blocks in addition to noninvasive positive pressure ventilation was effective in the management of a 74-year-old patient with impending respiratory failure resulting from excessive pain and narcosis following emergency laparotomy [50]. Similarly, Børglum et al. demonstrated that TAP blocks maybe an effective rescue therapy for patients with uncontrolled pain following major abdominal surgery [15]. The authors reported that their 4-point TAP block was effective in managing pain, decreasing opioid consumption, facilitating quicker postanesthesia care unit discharge, and improving mobilization. In addition, a growing number of reports suggest that TAP blocks may also be a safe alternative to neuraxial blockade in patients who are anti-coagulated, coagulopathic, or in patients who would not tolerate the hemodynamic sequelae often associated with profound neuraxial sympathectomy [29, 51, 52].

## 7. Complications

Complications of the TAP block are rare. To date, there are no published reports in the English language of local anesthetic toxicity following TAP blocks. Griffiths et al. reported a mean peak plasma ropivacaine level of 2.54 ± 0.75 mcg/mL using a total dose of 3 mg/kg to perform bilateral TAP blocks [53]. While this level is above previously established minimum toxic plasma levels of 2.2 mcg/mL, it is similar to levels achieved in other commonly utilized peripheral nerve blocks (e.g., 2.58 mcg/mL for axillary blocks). Kato et al. also suggested that toxic plasma levels maybe achieved when using 40 mL of 1% lidocaine [54]. Though direct intravascular injection of local anesthetics is very unlikely with TAP blocks, these studies do suggest that systemic toxicity is possible, and, as such, caution should be exercised throughout drug delivery.

Case reports of liver lacerations caused by right-sided TAP blocks can also be found in the literature. Farooq and Carey described a liver laceration after a landmark-based TAP block [55]. Upon laparotomy, the patient was subsequently found to have an enlarged liver that extended down to the iliac crest. As a consequence, the authors recommended routine palpation of the liver edge prior to landmark-based right-sided TAP blocks. Lancaster and Chadwick also reported a liver laceration after ultrasound-guided TAP block, which was likely as a result of failure to adequately visualize the needle during the procedure [56]. Furthermore, at least in theory, the spleen and kidneys are also at risk during TAP blocks. And although Jankovic et al. observed a TAP catheter in the peritoneal cavity upon surgical exposure of the abdomen for an open nephrectomy, no reports of injury to these organs were found during a thorough literature search [57].

While the likelihood of needle placement misadventures may be minimized with the proper use of ultrasound guidance, the potential complication of femoral nerve blocks (partial or complete) may not be completely avoided. The transversalis fascia comprises the fascial plane deep to the rectus abdominis muscles. This fascial plane is continuous with the *fascia iliaca*. Local anesthetic injected into the TAP can theoretically track along the transversalis fascia to the *fascia iliaca* and, in doing so, may block the femoral nerve and place the patient at risk of a fall.

## 8. Conclusions

The TAP block is an effective and safe adjunct to multimodal postoperative analgesia for abdominal surgery. Multiple studies have demonstrated its superiority over standard medical therapy for postoperative pain control. Limited data also suggest, that in select patient populations, TAP blocks/catheters may provide comparable analgesia as well as patient satisfaction to epidural therapy. However, the data is less encouraging for patients who receive intrathecal morphine during c-section, where the addition of TAP blocks does not appear to improve postoperative pain control. Nonetheless, it may be a good alternative strategy for patients who are highly sensitive to opioids.

Absolute contraindications to TAP blocks include patient refusal, soft tissue infection of the abdominal wall and skin, or abnormality at the needle insertion site. Coagulation status is an area of uncertainty with the TAP block and will require further investigation. Optimal dosing schemes (i.e., single shot versus catheter, intermittent versus continuous catheter infusions, type of local anesthetic, use of adjuvants) will also need to be determined. Moreover, there remains considerable debate over which type of TAP block provides the best coverage for specific surgeries. While many believe that the posterior approach is ideal for incisions below the umbilicus, that the subcostal block is best suited for upper abdominal procedures, and that a combined approach provides the greatest analgesic coverage, supporting data is conflicting at best. Well-designed and adequately powered studies are needed to address these clinically relevant questions.

## Figures and Tables

**Table 1:** List of published clinical studies on the use of TAP blocks. Single patient case reports have been excluded. (*) 20 patients were randomized to receive TAP block and spinal block with plain bupivacaine, while 20 patients were randomized to receive TAP block, bupivacaine spinal block, and intrathecal morphine. (**) Volume includes some local anesthetic used to perform ilioinguinal block in conjunction with TAP block. US-guided: ultrasound-guided. L-bupivacaine: Levobupivacaine.

Reference	Study sample	Local anesthetic	Block operator	Surgical procedure	Outcome
Sforza et al., 2011 [21]	14 cases 14 controls	10 mL/side of 0.5% bupivacaine with 10 mL/side lidocaine + 1 : 200,000 epinephrine	Surgical team	Abdominoplasty	Superior analgesia compared to IV/PO medications
Kadam and Moran 2011 [22]	15 cases 15 controls	8 mL/hr/side (continuous bilateral catheters) of 0.2% ropivacaine	Anesthesia team	Variety of upper and/or lower abdominal surgery	Noninferior outcome compared to epidural analgesia
Hivelin et al., 2011 [23]	15 cases 15 controls	1.5 mg/kg/side of 0.475% ropivacaine	Surgical team	Breast reconstruction with deep inferior epigastric perforator flap	Superior analgesia compared to IV/PO medications
McMorrow et al., 2011 [24]	40 cases 40 controls*	1 mg/kg/side of 0.375% bupivacaine	Anesthesia team	C-section	Inferior analgesia compared to intrathecal morphine
Mei et al., 2011 [25]	4 cases	20 mL/side** of 0.5% ropivacaine	Anesthesia team (US-guided)	C-section	Superior analgesia compared to local infiltration
Bharti et al., 2011 [17]	20 cases 20 controls	20 mL/side of 0.25% bupivacaine	Surgical team	Colorectal surgery	Superior analgesia compared to IV/PO medications
Børglum et al. 2011 [15]	25 cases	30 mL/side of 0.25% bupivacaine	Anesthesia team (US-guided)	Various upper and/or lower abdominal surgery	Significant reduction in pain and anticipated need for IV/PO analgesics
Niraj et al., 2011 [26]	29 cases 33 controls	1 mg/kg/side/8 hr (intermittent dosing through bilateral catheters) of 0.375% bupivacaine	Anesthesia team (US-guided)	Open hepatobiliary or renal surgery	Noninferior outcome compared to epidural analgesia
Aveline et al., 2011 [27]	134 cases 139 controls	1.5 mg/kg (unilateral block) of 0.5% L-bupivacaine	Anesthesia team (US-guided)	Open inguinal hernia repair	Superior analgesia compared to landmark-based ilioinguinal/iliohypogastric nerve block
Owen et al., 2011 [18]	16 cases 18 controls	20 mL/side of 0.25% bupivacaine	Surgical team	C-section	Superior analgesia compared to IV/PO medications
Gravante et al., 2011 [28]	51 cases	1 mg/kg/side of 0.5% bupivacaine	Surgical team	Abdominoplasty	Superior analgesia compared to IV/PO medications
Allcock et al., 2010 [29]	2 cases	20 mL/side bolus with 0.5% bupivacaine + 1 : 400,000 epinephrine followed by 8 mL/hr bilateral continuous infusion of 0.125% bupivacaine	Anesthesia team (US-guided)	Major thorax/abdominal trauma	Superior outcomes compared to expected results with IV/PO medications
Baaj et al., 2010 [30]	20 cases 20 controls	20 mL/side of 0.25% bupivacaine	Anesthesia team (US-guided)	C-section	Superior analgesia compared to IV/PO medications
Heil et al., 2010 [31]	3 cases	30 mL unilateral bolus of 1.5% mepivacaine followed by 8 mL/hr continuous unilateral infusion of 0.2% ropivacaine	Anesthesia team (US-guided)	Open inguinal hernia repair	Superior outcome compared to anticipated results with IV/PO medications
Chiono et al., 2010 [32]	33 cases	15 mL (unilateral block) of 0.33% ropivacaine	Anesthesia team (US-guided)	Iliac crest bone graft	Superior outcome compared to anticipated results with IV/PO medications
Araco et al., 2010 [19]	34 cases 41 controls	1 mg/kg/side of 0.5% bupivacaine	Surgical team	Abdominoplasty	Superior analgesia compared to IV/PO medications
Griffiths et al., 2010 [33]	32 cases 33 controls	15 mL/side of 0.5% ropivacaine	Anesthesia team (US-guided)	Gynecological malignancy surgery	No benefit in analgesia compared to IV/PO medications
Lee et al., 2010 [34]	50 cases	20 mL of 1% ropivacaine for unilateral blocks and 20 mL/side of 0.5% ropivacaine for bilateral blocks	Anesthesia team (US-guided)	Dermatomal coverage of subcostal versus posterior approach	Most cephalad dermatome of T8 by subcostal approach versus T10 by posterior approach
Kanazi et al., 2010 [35]	29 cases 28 controls	20 mL/side of 0.375% bupivacaine + 1 : 200,000 epinephrine	Anesthesia team (US-guided)	C-section	Inferior analgesia compared to intrathecal morphine
Mukhtar and Khattak 2010 [36]	10 cases 10 controls	20 mL (unilateral block) of 0.5% bupivacaine	Anesthesia team	Renal transplant	Superior analgesia compared to IV/PO medications
Conaghan et al., 2010 [37]	40 cases 34 controls	20 mL/side of 0.25% L-bupivacaine	Anesthesia team (US-guided)	Laparoscopic colorectal surgery	Superior analgesia compared to IV/PO medications
Araco et al., 2010 [38]	24 cases	1 mg/kg/side of 0.5% bupivacaine	Surgical team	Abdominoplasty	Superior outcome compared to anticipated results with IV/PO medications
Asensio-Samper et al., 2010 [39]	2 cases	20 mL (unilateral block) of 0.5% ropivacaine	Anesthesia team (US-guided)	Morphine pump implantation	Superior outcome compared to anticipated results with IV/PO medications
Costello et al., 2009 [40]	47 cases 49 controls	20 mL/side of 0.375 Ropivacaine	Anesthesia team	C-section	Noninferior outcome compared to intrathecal morphine
Jankovic et al., 2009 [41]	7 cases 35 controls	20 mL unilateral bolus of 0.375% L-bupivacaine followed by 10 mL/hr continuous unilateral 0.15% Bupivacaine infusion	Surgical team	Renal transplant	Superior outcome compared to IV/PO medications
Niraj et al., 2009 [42]	3 cases	20 mL/side/12 hr of 0.5% or 0.375% bupivacaine for bilateral catheters and 25 mL/12 hr of 0.5% bupivacaine for unilateral catheter	Anesthesia team (US-guided)	Upper and/or lower abdominal surgeries	Superior outcome compared to anticipated results with IV/PO medications
Belavy et al., 2009 [43]	23 cases 24 controls	20 mL/side of 0.5% ropivacaine	Anesthesia team (US-guided)	C-section	Superior outcome compared to IV/PO medications
Niraj et al., 2009 [44]	3 cases	1 mg/kg/side of 0.375% bupivacaine	Anesthesia team (US-guided)	Upper abdominal surgery	Superior outcome compared to anticipated results with IV/PO medications
Niraj et al., 2009 [45]	26 cases 26 controls	20 mL (unilateral block) of 0.5% bupivacaine	Anesthesia team (US-guided)	Open appendectomy	Superior outcome compared to IV/PO medications
El-Dawlatly et al., 2009 [46]	21 cases 21 controls	15 mL/side of 0.5% bupivacaine	Anesthesia team (US-guided)	Laparoscopic cholecystectomy	Superior outcome compared to IV/PO medications
Carney et al., 2008 [47]	24 cases 26 controls	1.5 mg/kg (max 20 mL)/side of 0.75% ropivacaine	Anesthesia team	Total abdominal hysterectomy	Superior outcome compared to IV/PO medications
McDonnell et al., 2008 [48]	25 cases 25 controls	1.5 mg/kg (max 20 mL)/side of 0.75% ropivacaine	Anesthesia team	C-section	Superior outcome compared to IV/PO medications
McDonnell et al., 2007 [12]	16 cases 16 controls	20 mL/side of 0.375% L-bupivacaine	Anesthesia team	Bowel resection	Superior outcome compared to IV/PO medications
O'Donnell 2006 [8]	12 cases	20 mL/side of 0.375% bupivacaine	Anesthesia team	Open retropubic prostatectomy	Superior outcome compared to anticipated results with IV/PO medications
